# A snapshot of cardiovascular diseases in Africa in the new millennium

**Published:** 2013-06

**Authors:** Andre Pascal Kengne, Bongani M Mayosi

**Affiliations:** Department of Medicine, University of Cape Town and South African Medical Research Council, Cape Town, South Africa; Department of Medicine, Groote Schuur Hospital and University of Cape Town, Cape Town, South Africa

The ageing of populations observed in many settings around the world extends to sub-Saharan Africa (SSA), where life expectancy at birth has increased by about 18 years between 1950 and 2010.^1^ It has been estimated that ageing of African populations will accelerate further in the coming decades, such that the gap in life expectancy with the world average will drop from 20 years in 2010, to only about 10 years by 2050. Lessons learned from other parts of the world suggest that changes in the age structure of populations with increasing proportions of elderly people, which is also known as ‘demographic transition’, is usually paralleled by changes in health profiles, which have been characterised as ‘epidemiological transitions’.^2^

Based on the Western models of epidemiological transitions, chronic non-communicable diseases (NCDs) replace infectious diseases as the major cause of death and disability during the demographic transition. Several lines of evidence suggest that chronic NCDs are gaining ground in Africa; however, this against a background of highly prevalent infectious diseases, some of which have chronic patterns, which are still contributing a very significant proportion of deaths on the continent. These competing disease profiles have been detrimental for chronic diseases; resulting in knowledge generation and translation, policy formulation and implementation, and health service development for chronic NCDs to severely lag behind those for infectious diseases in Africa.

A major challenge in appraising the true magnitude of chronic NCDs and on-going efforts to prevent and control them in Africa has been the lack of locally relevant scientific evidence.^3^ There is, however, the suggestion that the situation is rapidly improving, with the growing number of high-quality scientific publications that are emerging on NCDs in Africa.^4^ This was already apparent in December 2005, when the leading world journal of cardiovascular diseases, *Circulation*, undertook to publish a dedicated series of scientific articles on cardiovascular diseases (the leading chronic NCDs) in sub-Saharan Africa.^5^ This series of nine scientific articles plus an editorial had the particularity of been driven essentially by African researchers who were able to rigorously build on personal experience and published data at the time, and provide a comprehensive and representative picture of cardiovascular disease in SSA.

The success of the articles in the *Circulation* series,^5^ as measured by the large number of citations attached to each of them, attests to the influential role the initiative of *Circulation* has played within less than 10 years in shaping the landscape of cardiovascular diseases in SSA. However, this series was mostly based on evidence from the last century, while more evidence is emerging in the context of improved access to care for major infectious diseases such as HIV infection and tuberculosis, and increasing awareness on chronic NCDs in Africa.^6^ Regular updates are therefore needed to carry the wave initiated by *Circulation* in 2005 across time.^5^ It is exactly what a group of dedicated scientists, including some who were already involved in the 2005 initiative, have successfully achieved.

In a series titled ‘Cardiology in Africa 2013 Update’ published online on 15 May 2013 in *Heart*, a leading international cardiovascular journal from the *British Medical Journals’* group, a comprehensive overview is provided on recent developments on cardiovascular diseases in Africa, and strategies to improve the prevention and control discussed by leading scientists with established research experience on CVD in Africa. The series has at least two distinctive features. One is the period covered by the included studies, which does not extend beyond the last 10 years, therefore allowing the series to project an unclouded picture of CVD in Africa in the early 21st century.

The second feature of the series is the nearly equal coverage given to diseases of modern lifestyle which are often the major focus when approaching CVD from a global perspective, and the neglected CVDs, which are rather rare elsewhere, yet remain a major challenge in Africa. For instance, in one of the articles dedicated to endomyocardial fibrosis (EMF), a condition described for the first time in Uganda in 1938,^7^ Drs Mocumbi and Falase^8^ provide findings confirming that three-quarters of a century from the first description, EMF has remained a frequent but neglected CVD in Africa. As a consequence, little is known on the mechanisms and treatment of the disease, which is still associated with a poor prognosis.

Recent advances on acute heart failure and cardiomyopathies are covered in another article in the series by Sliwa and Mayosi.^9^ Reliable and robust evidence on acute heart failure have recently emerged from a multicentre, cross-continental study, showing that 90% of cases of heart failure in Africa can be attributed to hypertension, rheumatic heart disease and cardiomyopathy, which is at variance with findings from the West where heart failure is largely due to coronary heart disease.^10^ Furthermore, heart failure in Africa is a disease of young people, with a similar high early mortality rate as found elsewhere, and the uptake of evidence-based therapies for heart failure by healthcare providers in Africa is still below optimal.

The epidemiology of cardiomyopathy, a leading cause of heart failure in Africa, has been refined in recent studies in Africa. Findings from those studies are informing the design and implementation of further investigations into the pathogenesis and management of cardiomyopathies on the continent.^9^

Cardiovascular diseases in specific populations within Africa are covered in two articles in the series. In their article on CVD in people with HIV, Syed and Sani^11^ shed light on the contrast in the spectrum of CVD among people with HIV in the West and those in Africa. While improving survival among people with HIV infection on antiretroviral therapy in the West has been associated with premature occurrence of CVD of modern lifestyle, the spectrum of CVD among those in Africa is still dominated by cardiomyopathies, pericardial diseases and pulmonary hypertension. While improving the detection and management of the latter is critical, it is also very important for healthcare providers in Africa to remain watchful as the CVD profile in people with HIV may change with time, as many people will live longer on antiretroviral therapies.

Childhood CVDs are discussed by Drs Zühlke, Mirabel and Marijon^12^ in an article demonstrating that these conditions are largely dominated by rheumatic heart diseases (RHD) and congenital heart disease (CHD). They tend to occur at much higher rates in Africa than in other parts of the world, where they are associated with poor prognosis resulting from delayed diagnosis and treatment. While appropriate prevention and/or treatment measures for these conditions exist, their uptake in Africa is still inappropriate. In spite of the resource constrains, implementation projects for early diagnosis of CHD, followed by corrective surgery are underway in many parts of the continent.^13^ Such efforts for RHD are limited by the many uncertainties regarding optimal age for screening, the appropriate screening tool, and course of action following screening.^14^

Cardiovascular diseases of a modern lifestyle and major risk factors are covered in four articles in the series. In one of these four, myocardial infarction and stroke in Africa are revisited by Ntsekhe and Damasceno.^15^ Their report suggests that ischaemic heart disease is still a relatively modest contributor of CVD in Africa; however, the burden of risk factors for atherosclerosis is rapidly increasing across the diversity of rural and urban settings in Africa.

Stroke on the other hand is now an established major cause of premature mortality and disabilities in Africa, resulting primarily from hypertension and less from atherosclerosis. Hypertension is covered in great detail by Ogah and Rayner,^16^ showing that hypertension is one of the most investigated cardiovascular risk factors in Africa. Since 2007 when the last comprehensive overview of hypertension studies in Africa was published,^17^ at least 38 new studies have been reported, mostly from urban Africa. Increasingly high prevalences of hypertension continue to be reported against a background of low rates of detection, treatment and control.

The last articles of the series are two companion articles by Kengne and collaborators,^18^,^19^ focusing on diabetes and obesity. They report that diabetes incidences are on the rise in Africa, essentially paralleling those for obesity, but studies to characterise them have been less than optimal. The care of diabetes largely remains suboptimal in most countries, which are ill-prepared to face the control and prevention of diabetes, as the costs of caring for the condition pose a challenge to most local economies. Moreover, translation strategies to prevent and control diabetes and obesity in Africa are still to be evaluated.^18^,^19^

Altogether, the eight articles provide a timely and comprehensive overview of the evidence currently available to carry the fight against cardiovascular diseases into the second decade of the 21st century and beyond. In the accompanying editorial, Bongani Mayosi, the series editor, shows strong support for the 10 key population-level interventions of proven cost-effectiveness that are well-suited to the low-income settings of African countries, as the appropriate recipes for preventing cardiovascular diseases from compromising the long-expected and gradually experienced economic development of the African continent.^20^ Concerted action will be needed to integrate the ‘10 best buys’ for the prevention and treatment of heart disease, diabetes and stroke in the national plans for action on NCDs in African countries.

## References

[1] (2012). United Nations, Department of Economic and Social Affairs, Population Division, Population Estimates and Projections section.

[2] Omran AR (1998). The epidemiologic transition theory revisited thirty years later.. World Health Stat Q.

[3] Kengne AP, Ntyintyane LM, Mayosi BM (2012). A systematic overview of prospective cohort studies of cardiovascular disease in sub-saharan africa.. Cardiovasc J Afr.

[4] Jones AC, Geneau R (2012). Assessing research activity on priority interventions for non-communicable disease prevention in low- and middle-income countries: A bibliometric analysis.. Glob Health Action.

[5] Opie LH, Mayosi BM (2005). Cardiovascular disease in sub-Saharan africa.. Circulation.

[6] Echouffo-Tcheugui JB, Kengne AP (2012). A United Nation high level meeting on chronic non-communicable diseases: Utility for Africa?. Pan Afr Med J.

[7] William AW (1938). Heart disease in the native population of uganda. Part 1. Syphilitic heart disease.. East Afr Med J.

[8] Mocumbi AO, Falase AO (2013). Recent advances in the epidemiology, diagnosis and treatment of endomyocardial fibrosis in Africa.. Heart.

[9] Sliwa K, Mayosi B (2013). Recent advances in the epidemiology, pathogenesis and prognosis of acute heart failure and cardiomyopathies in Africa.. Heart.

[10] Damasceno A, Mayosi BM, Sani M, Ogah OS, Mondo C, Ojji D (2012). The causes, treatment, and outcome of acute heart failure in 1006 africans from 9 countries.. Arch Intern Med.

[11] Syed FF, Sani MU (2013). Recent advances in HIV-associated cardiovascular diseases in africa.. Heart.

[12] Zuhlke L, Mirabel M, Marijon E (2013). Congenital and rheumatic heart disease in africa: Recent advances and current priorities.. Heart.

[13] Awori MN, Ogendo SW, Gitome SW, Ong’uti SK, Obonyo NG (2007). Management pathway for congenital heart disease at Kenyatta National Hospital, Nairobi.. East Afr Med J.

[14] Zuhlke L, Mayosi BM (2013). Echocardiographic screening for subclinical rheumatic heart disease remains a research tool pending studies of impact on prognosis.. Curr Cardiol Rep.

[15] Ntsekhe M, Damasceno A (2013). Recent advances in the epidemiology, outcome, and prevention of myocardial infarction and stroke in subsaharan africa.. Heart.

[16] Ogah OS, Rayner BL (2013). Recent advances in hypertension in africa.. Heart.

[17] Addo J, Smeeth L, Leon DA (2007). Hypertension in sub-Saharan Africa: A systematic review.. Hypertension.

[18] Kengne AP, Echouffo-Tcheugui J, Sobgnwi E, Mbanya JC (2013). New insights on diabetes mellitus and obesity in africa. Part 1: Prevalence, pathogenesis and comorbidities.. Heart.

[19] Kengne AP, Echouffo-Tcheugui J, Sobgnwi E, Mbanya JC (2013). New insights on diabetes mellitus and obesity in Africa. Part 2: Prevention, screening and economic burden.. Heart.

[20] Mayosi B (2013). The 10 ‘best buys’ to combat heart disease, diabetes and stroke in Africa.. Heart.

